# Cultivation Using Coir Substrate and P or K Enriched Fertilizer Provides Higher Resistance to Drought in Ecologically Diverse *Quercus* Species

**DOI:** 10.3390/plants12030525

**Published:** 2023-01-23

**Authors:** Barbara Mariotti, Sofia Martini, Sabrina Raddi, Francesca Ugolini, Juan A. Oliet, Douglass F. Jacobs, Alberto Maltoni

**Affiliations:** 1 Dipartimento di Scienze e Tecnologie Agrarie, Alimentari, Ambientali e Forestali—DAGRI, Università di Firenze, Via San Bonaventura 13, 50145 Firenze, Italy; 2 Istituto per la Bioeconomia, Consiglio Nazionale delle Ricerche, Via Madonna del Piano 10, 50019 Sesto Fiorentino, Italy; 3 Departamento de Sistemas y Recursos Naturales, Universidad Politécnica de Madrid, Ciudad Universitaria s/n, 28040 Madrid, Spain; 4 Department of Forestry and Natural Resources, Hardwood Tree Improvement and Regeneration Center, Purdue University, West Lafayette, IN 47907, USA

**Keywords:** water stress, growing media, fertilization, seedling survival, height growth, physiological traits, morphological traits, *Quercus robur*, *Quercus pubescens*, *Quercus ilex*

## Abstract

Nursery cultivation practices can be modified to increase resistance to water stress in forest seedlings following field establishment, which may be increasingly important under climate change. We evaluated the morphological (survival, growth) and physiological (chlorophyll fluorescence, leaf water potential) responses to water stress for three ecologically diverse *Quercus* species (*Q. robur*, *Q. pubescens*, and *Q. ilex*) with varying traits resulting from the combination of growing media (peat, coir) and fertilization (standard, P-enriched, K-enriched). For all species under water stress, seedlings grown in coir had generally higher growth than those grown in peat. Seedlings fertilized with P performed better, particularly for survival; conversely, K fertilization resulted in inconsistent findings. Such results could be explained by a combination of factors. P fertilization resulted in higher P accumulation in seedlings, while no K accumulation was observed in K fertilized seedlings. As expected, the more drought-sensitive species, *Q. robur*, showed the worst response, while *Q. pubescens* had a drought resistance equal or better to *Q. ilex* despite being classified as intermediate in drought resistance in Mediterranean environments.

## 1. Introduction

Under the threat of climate change, one of the most adverse pressures on natural ecosystems is drought and/or heat stress [1,2,3]. In recent decades, unusual frequencies and durations of dry periods in combination with high temperatures have been observed especially in the Mediterranean basin [4,5]. Nowadays, such conditions are affecting regeneration, growth, and productivity of the Mediterranean forests [1,6], modifying forest composition and structure [7,8], and reducing sensitive species survival [9,10] and biodiversity [11,12]. The northern Mediterranean areas are characterized by limited periods of drought, mainly occurring in the middle of the summer. Nevertheless, progressive severity and unpredictability of such events are increasing, with dry periods occurring also in other non-typical seasons such as spring and fall [13]. Such changes can inhibit the success of forest restoration and reforestation projects [14,15]. In the northern Mediterranean basin, spring and to a lesser extent fall, are traditionally transplanting seasons, thus newly outplanted seedlings could unexpectedly suffer from the lack of optimal conditions in such a variable phase [16,17]. Moreover, in several Mediterranean countries, post-planting practices designed to promote seedling survival are often not targeted to site-limiting factors, especially in degraded areas [18,19].

Nursery cultivation practices can be modified to increase forest seedling drought hardiness and reduce transplanting shock [20,21,22,23]. Although functional traits related to transplanting performance are species specific [15,24,25], these traits are strongly influenced by nursery practices, including seed selection, methods of cultivation, and transplanting practices [26,27]. Among them, seedling drought preconditioning by reducing irrigation promotes morphological and physiological responses in seedlings that make them more resistant to drought [22,28,29,30,31].

Beyond drought hardening, other nursery practices such as the type of substrate and fertilization [21,32] can affect seedling morphology [23,33,34,35] and physiological attributes [22,36,37,38,39,40,41]. Nutrient availability during cultivation augments the mineral reserves of seedlings that can be remobilized to fuel new growth after planting [42,43]. Essential nutrients play key roles in functional attributes related to seedling establishment [44] and can increase the speed at which a seedling overcomes planting stress [40]. Hence, fertilization can be used to improve seedling quality [45,46] and performance when transplanted into the field [47]. Many authors studied such effects with nitrogen (N) fertilization [28,40,44,48,49], while phosphorous (P) and potassium (K) fertilization have been less studied. P plays a key role not only in biochemical processes, such as storing and transferring energy, photosynthesis, enzyme formation and regulation [50,51], but also in morpho-functional traits of the root system [23,52]. Thus, P can increase plant capacity to face drought associated with enhanced root growth, stomatal conductance, nitrate reductase and anti-oxidative enzyme activities, leaf area, photosynthetic rate, and water relations [45,53,54]. K is related to a plethora of physiological processes [55], as one of the key important osmolyte in plants [56,57]. It assures an adequate turgor pressure for cell expansion and growth and enhances tolerance to drought and high light [58]. However, only a few studies have shown a significant (but weak) positive relationship between K concentration and outplanting performance [28,59,60]. Additional studies are needed to better understand the role of these two nutrients in post-planting performance, especially under threats of drought intensity increase.

The most commonly used substrate for nursery production is a mixture of organic (mostly peat [61,62]) and mineral compounds (e.g., perlite, vermiculite, or expanded clay). New solutions for implementing sustainable processes in nurseries have promoted the use of renewable and more sustainable materials than peat [63,64,65]; coconut fiber (coir) is one of the most common among these [62]. In general, substrates can influence seedling morpho-physiological traits [66,67,68,69]; however, relatively few studies have compared functional responses to post-planting drought as affected by alternative growing media formulations.

In this framework, we performed a water stress resistance experiment under semi-controlled conditions in a greenhouse, testing six stocktype combinations resulting from two substrates (peat and coir, *Pe* and *Co*, respectively)) and three fertilization treatments (nursery standard control, P enriched, K enriched). We analyzed the response of these stocktype combinations to three levels of post-transplant water stress after a period of undisturbed growth for three species of *Quercus* (*Q. robur* L., *Q. pubescens* Willd., *Q. ilex* L., hereafter QR, QP, and QI, respectively). These species are commonly used in reforestation or forest restoration projects in the Northern Mediterranean area [19,70,71,72], following a recognized gradient of drought tolerance: from the most resistant to xeric environments, the evergreen oak *Q. ilex* L., which is drought tolerant, to the most suited to temperate areas and more vulnerable to water stress, *Q. robur* L. [73,74]. We anticipated that the varying seedling stocktypes of *Quercus* spp. would respond differently to water stress (in terms of survival, growth, and physiological indices) as a function of varying drought tolerance; specifically, we hypothesized that (1) the growing media characteristics would affect water relations, with the peat-based substrate resulting in lower levels of water stress; (2) nursery fertilization would affect the response, with higher K and P playing a role in a higher resistance as they are key elements in physiological processes related to drought resistance and vitality.

## 2. Results

For the vast majority of the variables, seedlings under medium stress provided results not significantly different from the control; thus, this treatment was excluded from the analysis. When medium stress analysis provided relevant information to data discussion, this was reported in the text.

### 2.1. Water Stress Effects on Seedling Survival, Leaf Mortality, Plant Height, and Recovery after Irrigation

In all species, the percentage of seedlings with dry shoot at T_1_ and mortality T_rec_ occurred only under high water stress (HS), whilst under control (C) and medium stress, no mortality (0%) occurred in all stocktypes. At both T_1_ and T_rec_, the substrate did not affect either percentage of seedlings with dry shoot or mortality (Table 1), while fertilizations affected them in *Q. robur* and in *Q. pubescens* though with different results (Table 1 and Table 2): in the case of *Q. robur*, K-enriched fertilization resulted in higher mortality and P-enriched fertilization increased survival of *Q. pubescens*. No statistical differences between mortality rates in different fertilizations were observed in *Q. ilex*, though a marginally higher value in P-enriched fertilization plants was found (Table 1 and Table 2). Neither dry shoot at T_1_ nor survival at T_rec_ were influenced by substrate x fertilization interactions (Table 1). 

At T_1_, all leaves on seedlings under *C* were alive, while 53% of the leaves died under *HS*. *Q. robur* had the highest leaf mortality (78%) followed by *Q. ilex* (50%) and *Q. pubescens* (30%). Seedlings grown on coir were able to retain a larger fraction of live leaves (not significant), especially in *Q*. *pubescens* (91%; *Q. ilex* 69%, *Q. robur* 56%). With regard to fertilization, *Q. pubescens* and *Q. ilex* seedlings grown under enriched *K* or *P* nutrition in *HS* conditions had a higher fraction of live leaves as compared to *St*: *K* promoted leaf survival in both oak species (100% and 86% in QP and QI, resp.; *p* ≤ 0.01), while *P* only in *Q*. *ilex* (67%; *p* ≤ 0.01). Differently, *Q. robur* seedlings grown in K-enriched fertilization showed almost complete (94% on total) leaf mortality, much higher (*p* ≤ 0.01) than in *P* (73%) or *St* (69%) fertilizations. 

**Figure 1:**
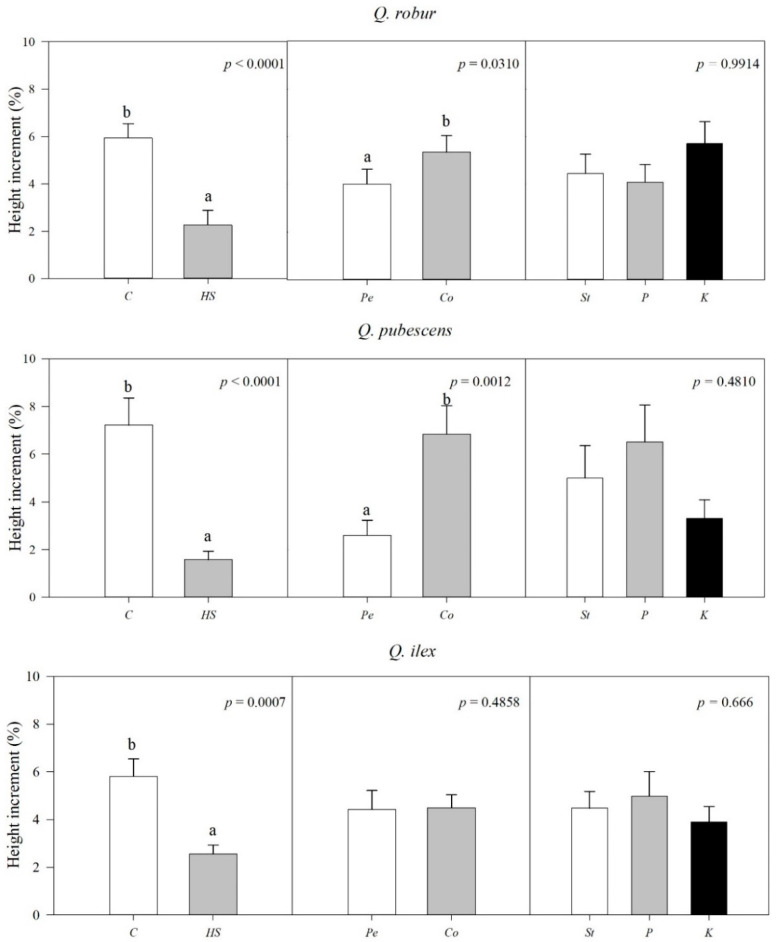
Mean values (and SE) of seedlings’ percent height increment (I%) at T_1_ as affected by water stress (**left**), substrate (**center**), and fertilization (**right**). P values from multifactorial ANOVA are shown on top of each sub-figure. Lowercase letters indicate homogeneous groups (*p ≤ 0.05*). *C*, *HS*: control and high water stress; *Pe*, *Co*: peat and coir substrate; *St*, *P*, *K*: standard, P- and K-enriched fertilization.

The size of the seedling at the beginning of the experiment (Table 3) was significantly higher in seedlings grown in peat than in coir and in *K* fertilization in all species, excluding *Q. ilex*, for which fertilization did not have a significant effect. Percent height increment (I%) was strongly influenced by water stress; actually, a significant effect was already observed after one week of treatment (*p* ≤ 0.001, data not shown). In fact, at T_1_ in all species, seedlings grew significantly less under high water stress than in control. Despite the general trend in which the higher the water stress, the lesser I% (*C* > *MS* > *HS*), I% significantly differed between the three water regimes only in *Q. robur* (*p* < 0.001). Comparing *C* and *HS* (Figure 1), in *Q. pubescens*, I% was significantly reduced under high stress conditions with respect to control (*HS* about 4 times lower than *C*), while in *Q. ilex*, I% under *MS* was similar to I% in both HS or *C*. *Q. robur* and *Q. pubescens* seedlings formerly grown in coir had significantly higher I% than those grown in peat. Conversely, fertilization did not influence seedling growth response. Interactions between and among factors (water stress, substrate, fertilization) were never significant (data not shown). In terms of absolute values of height increment, the majority of live seedlings under *HS* (59.6% in QR, 73.8% in QP, and 38.7% in QI) showed a negligible (<1 cm) or null height growth between T_0_ and T_1_, while in *C*, the mean values were 3.7 ± 3.4 cm, 2.3 ± 2.9 cm and 3.5 ± 3.5 cm for *Q. robur*, *Q. pubescens*, and *Q. ilex*, respectively. ANOVA performed on increment absolute value confirmed the results of I% (data not shown), in both variables, interactions were not significant.

### 2.2. Leaf Water Potential

**Figure 2:**
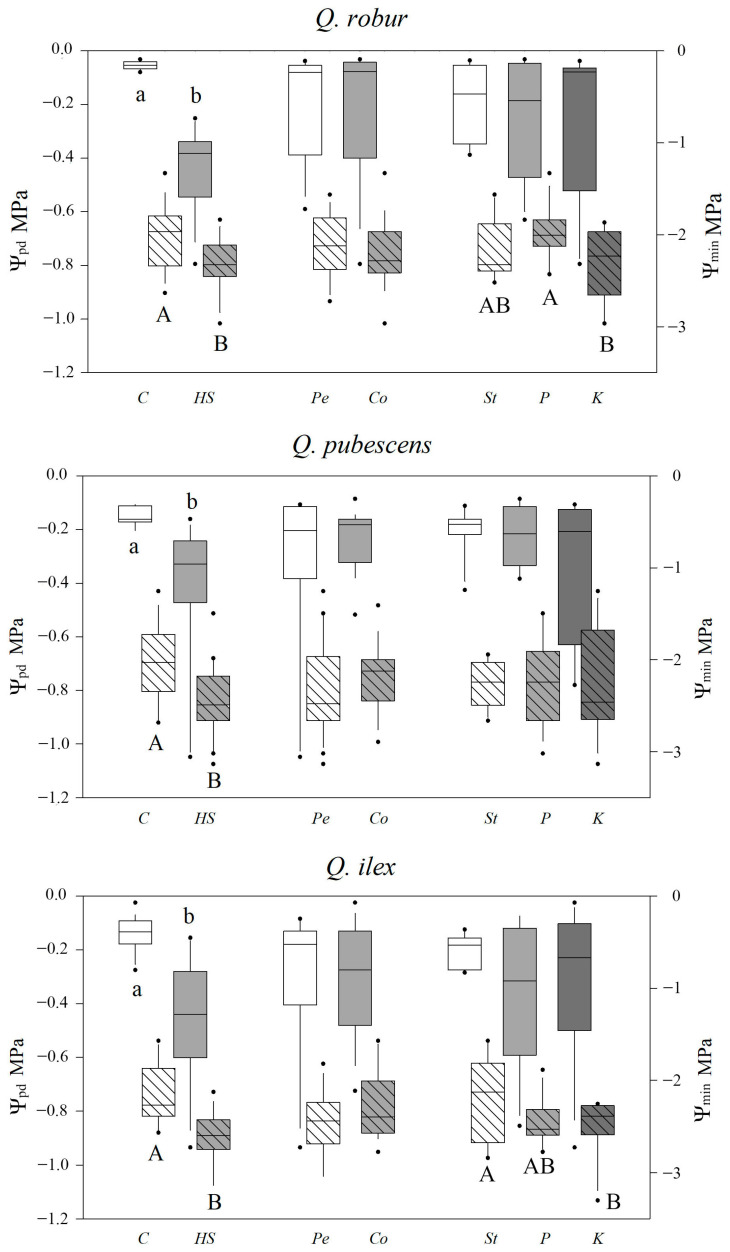
Boxplots of predawn (Ψ_pd_, blank boxplot) and midday leaf water potential (Ψ_min_, stripped boxplot) at T_1_. Lowercase and capital letters indicate homogeneous groups (*p* ≤ 0.05) for Ψ_pd_ and Ψ_min_, respectively. Levels as affected by studied factors: *C*, *HS*: control and high water stress; *Pe*, *Co*: peat and coir substrate *St*, *P*, *K*: standard, P- and K-enriched fertilization. Boxplot limits correspond to 25th and 75th percentiles, line is the median, whiskers represent expected data variability outside the upper and lower quartiles, points are outliers.

At T_0_, seedlings of all species had Ψ_pd_ near to zero, while Ψ_min_ ranged between QI (−2.23 ± 0.31 SD MPa) and QR (−1.05 ± 0.32 SD Mpa) values, without significant differences between growth substrates or fertilizations. At the end of the water stress experiment in all species, both Ψ_pd_ and Ψ_min_ decreased in *HS* with respect to *C* (*p* ≤ 0.01) (Figure 2). No significant effect was observed between substrates on both Ψ values. Instead, fertilization had an effect on Ψ_min_ in for *Q. robur* (*K* < *P*; *p* ≤ 0.05, and *St* not different from both) and *Q. ilex* (*K* < *St*; *p* ≤ 0.01, and *P* not different from both). Interactions were not significant in all comparisons.

### 2.3. Chlorophyll Fluorescence

**Figure 3:**
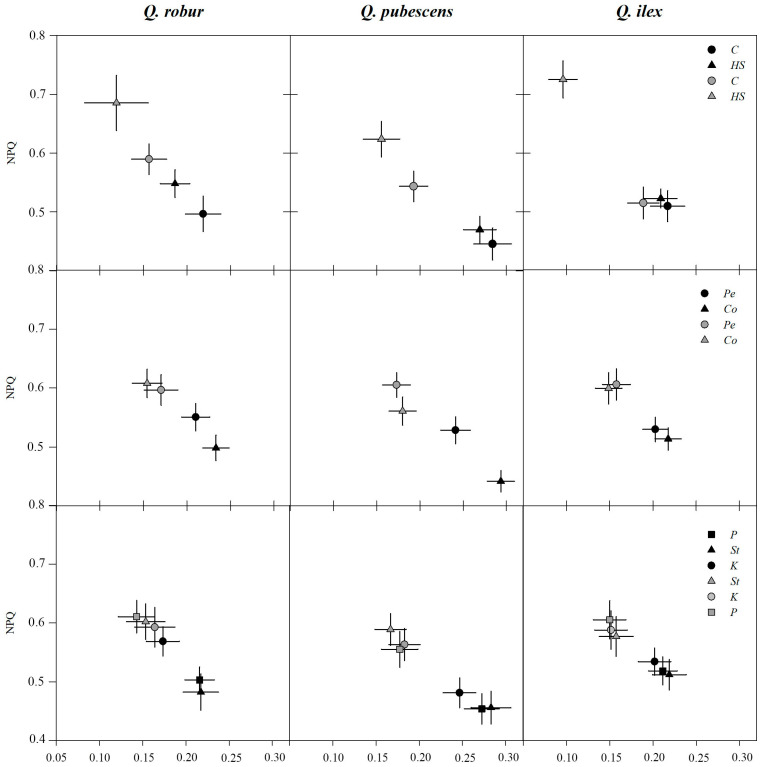
Fluorescence quantum yields (Φ_II_ and Φ_NPQ_; mean and CI_95%_ bars) at T_0_ (black symbols) and T_1_ (grey symbols) for water stress (**top**), substrate (**mid**), and fertilization (**bottom**). When CI_95%_ bars overlap data are not significantly different. Levels: *C*, *HS*: control, high water stress; *Pe*, *Co*: peat and coir substrate *St*, *P*, *K*: standard, P-enriched, K-enriched fertilization.

Φ_II_ and Φ_NPQ_ yields were negatively related (Figure 3). Before water-stress onset (T_0_), oaks had higher Φ_II_ when formerly grown on coir than on peat, counterbalanced by a decrease in Φ_NPQ_. *Co* effect on Φ_II_ was more marked in *Q. pubescens* (+22%) than in *Q. robur* (+12%) or *Q. ilex* (+8%). At T_0_, fluorescence yields did not differ in P-enriched fertilization on *St* in any of the three oaks species, while Φ_II_ was lower in K-enriched fertilization (respect to *St*) both in *Q. robur* (−20%) and *Q. pubescens* (−13%); such reduction was matched by comparable Φ_NPQ_ increases. In addition, a significant seasonal trend was observed in all the species under well-watered conditions (*C*) with Φ_II_ decrease and Φ_NPQ_ increase in mid-July (T_1_), confirming the necessity of *C* as a reference term. Under well-watered conditions, *Q. pubescens* showed the highest decrease between T_0_ and T_1_ in Φ_II_ of about −36%, followed by *Q. robur* (−29%) and *Q. ilex* (−25%). Such reductions were matched by Φ_NPQ_ increases (QR: +19%; QP: +25%; QI: +13%). 

At T_1_ under *HS*, *Q. robur* and *Q. pubescens* showed a significant Φ_II_ reduction (−22% than *C*) together with Φ_NPQ_ increase (+16% *C*) (Figure 3). Φ_II_ progressively and significantly decreased in *Q. ilex* under water stress, reaching values −49% (*HS*) lower than *C*, while Φ_NPQ_ increased by +41%, respectively. At T_1_, yields did not differ between substrates and fertilizations, except for a few exceptions: we observed lower Φ_NPQ_ for QP in *Co* (−9% *Pe*) and in *P* (−6% *St*) and higher Φ_II_ for QR in *K* (+14% *St*). GLM analysis showed a significant interaction for Φ_NPQ_ between *Sub × Fert* only for QR in *C* at T_1_ (*p* ≤ 0.02), where *CoSt* had a lower Φ_NPQ_ of 0.47 than in *PeSt*, *PeP*, or *CoP* ranging from 0.63 to 0.68.

In each oak species, at T_1_, the performance index on absorption basis (PI_ABS_)—which is the performance index for energy conservation of photons absorbed by PSII, through the electron transport chain, from PSII to PSI—was lower under high water stress than in control (Table 4), in a significant way for *Q. robur* and *Q. ilex*. Regarding substrates, seedlings formerly grown on coir showed higher PI_ABS_ than on peat at both T_0_ and T_1_, although significantly different only in *Q. pubescens*. Instead, after water stress (T_1_), PI_ABS_ was similar among all fertilization treatments. 

## 3. Discussion

Stress resistance in terms of shoot dryness and survival was influenced by mineral nutrition: without changing *N* supply—as in *P*-enriched fertilization—an increased dose of *P* was tendentially positive for surviving and recovering from severe water stress of *Q. ilex* and, with strongest effects, *Q. robur*, the most sensitive species to drought. This result could be related to the generally higher concentration of phosphorus in the plant tissues in P-enriched fertilization during the nursery cultivation than in the other two fertilizations [69]. Sardans et al. [75] found that P can improve the survival rate in *Q. ilex*, and it is acknowledged that P can positively influence physiology and performance in Mediterranean species [44,45], affecting—among others—aspects related to electron transport to photosystem I, and thus, productivity [76]. In contrast, higher K supply generally brought inconsistent results: a positive effect was clearly observed in *Q. pubescens*, while high mortality was observed in *Q. robur*. According to Haldimann et al. [77], *Q. pubescens* can reorganize the photosynthetic apparatus to maintain the functional capacity—despite premature leaf mortality—ensuring a rapid recovery under co-occurring conditions of harsh drought and high temperature, and solar irradiance, and this could explain the excellent performance of this species in this experiment. During nursery cultivation, K-enhanced fertilization promoted height and chlorophyll fluorescence, although *K* concentration in all plant tissues was comparable to that found in the other fertilization treatments [69]. This suggests that this element played a role in the growth of seedlings at the nursery stage [78,79,80], but to a lesser extent in water stress resistance after transplanting. Moreover, in our case, the poor performance in *Q. robur K*-treated seedlings could be explained by the high positive effect of K on shoot size [69] which was maintained to the beginning of the water stress, with effects on the water status that could negatively affect growth and survival of bigger seedlings or even promote cavitation [81]. Bigger seedlings may suffer more than smaller seedlings when faced with a restricted container volume for root exploration [82,83]; the container can differ from the potentially higher amount of water uptake that could occur in the field where roots can explore a larger soil volume to compensate for the water deficit. However, in our case, during the water stress, we maintained a constant substrate VWC threshold by daily irrigation, providing the same VWC conditions to all seedlings. The bigger seedlings may have been more likely to be affected by root confinement, i.e., from a non-natural development or deformation in the container, which could have promoted some differences in water uptake [84,85], than from volume constraints. Indeed, according to studies on *Pistacia lentiscus* and *Pinus canariensis* [52,86], small seedlings—despite lower root growth—could promote a higher short-term survival when planted in dry soils; thus, our results seemed to be aligned with such observations. A recent meta-analysis [87] and other studies [17,22] observed that some species, especially angiosperms, perform better in dry sites with larger planted seedlings, suggesting that higher water stress due to greater transpiration could not counteract the benefits due to higher shoot and root growth, such as an increased competitive capacity and stress avoidance related to a larger root system. Notably, our results followed a strong, intense period of water stress about two months after full irrigation; thus, we cannot speculate about an entire growing season after outplanting in the field. However, size itself can only partially explain the lower drought resistance of the seedlings under K-enriched fertilization. In fact, P-enriched fertilization performed better than standard fertilization even though the size at the end of nursery cultivation was higher [69], especially in *Q. robur.* Previous studies with this experiment showed that P-enriched fertilization promoted a higher number of green vs. dry leaves than other fertilizations [88].

Fertilization did not affect plant growth under water stress. Both extra fertilizations performed similarly to the standard even though height increment turned out to be the most sensitive parameter to water stress, since it responded very rapidly to water shortage in all species, as reported for *Q. robur* by Picon et al. [89]. In our case, both mild and severe water stress negatively affected height growth from the first week (data not shown), with more marked effects in *Q. robur* and *Q. pubescens.* Drought may induce turgor loss within hours [90], followed by a partial growth recovery, as observed in our experiment particularly in these two species. The recovery might be due to the solute accumulation [91], the activation of an effective plant protection machinery (e.g., in *Q. robur* by [92], in *Q. pubescens* by [77]), and the reduction of root elongation [93].

Conversely to fertilization, in all the studied species, the substrate did not have any effect on shoot desiccation and survival under water stress, even though seedlings grown in coir generally performed better than peat. Substrate affected the growth of transplants under highly severe stress conditions; coir was more effective than peat to sustain the growth of *Q. robur* and *Q. pubescens*. Such performance could be likely due to the greater development of roots on such substrate, i.e., a lower shoot-to-root ratio [69] and to generally smaller seedlings [69,94]. Although coir is known to have lower cation exchange capacity than peat [95], the material can be rich in K [62,96,97]. However, K content can vary widely according to the provenance and the treatments to make it usable as substrate [97,98]. An analysis requested by the nursery company that hosted this experiment (Vannucci Piante, Quarrata, Italy) on the same coir commercial product two years after this study showed that the K concentration of pure coir was 131 mg/l, which is low compared with that reported by Evans et al. [98]. A study on *Q. rubra* seedlings suggests that a substrate with a higher coir content stimulates root growth in detriment of the shoot [99]. If the hypothesis was confirmed, the use of coir-based substrates in drought-hardening techniques could be considered.

Leaf physiological measurements and physiological parameters related to photosynthetic activity generally agreed with survival and leaf desiccation; a clear drought effect was observed; watering regime was the most influential factor, while nursery cultivation effects occurred only in specific cases. The species showed acclimation responses to water stress following their ecological behavior, with *Q. robur* being more sensitive than the other oaks [73]. Predawn water potential reflects plant water status, which, in case of disequilibrium between plant and soil, may differ from soil water potential [100]; predawn water potential values under severe water stress were about −0.4 MPa and higher than the minimum values reported for these oak species in other studies, e.g., [101,102,103], but in line with other drought trials [97,104]. Oak transpiration was found to be more related to the hydraulic conductance of the soil–plant continuum than to soil Ψ [105], which means that stomata respond to the sole decrease in the root or plant hydraulic conductance, without the need for a decrease in soil water potential.

According to the literature (e.g., [106]) drought stress can favor a reduction in chlorophyll content due to the damage in the structure of the chloroplast membrane suggesting a reduction in photosynthetic capacity. Indeed, in our study, the functionality of the photosystem II (expressed by Φ_II_) decreased and confirmed expected values for the two deciduous oak species (*Q. pubescens*, −19%; *Q. robur*, −24%). The higher drop in *Q. ilex* Φ_II_ (−49%) is in line with different strategies between deciduous and evergreen broadleaves, and particularly oaks, in facing drought under high light and temperature [73], with greater photoprotective energy dissipation in the evergreens [107]. Here, *Q. ilex* showed high sensitivity to drought by decreasing Φ_II_ (and increasing Φ_NPQ_) as reported by [108], and higher tolerance to mid-July high temperatures as compared to the other two species, as it only slightly showed a down-regulating photochemical yield under full-irrigation. The effect of substrate on decreasing Φ_II_ (and increasing Φ_NPQ_) was evident only in *Q. pubescens*, where plants grown in coir performed better than in peat (*Co* vs *Pe*: 4% of Φ_II_, −9% of Φ_NPQ_), while no differences were due to different nursery fertilization. Similar results were provided by PI_ABS_, an index of the overall electron flow through PSII functionality, which was rapidly (already after one week—data not shown) influenced by water availability, confirming its sensitivity to drought stress [109,110] and particularly to severe water stress [111,112,113].

As expected, *Q. robur*, a typical tree of the Central Europe floodplain mixed forests, drought-sensitive species [114] confirmed its lower resistance under severe water stress [115,116,117,118]. According to [73], among broadleaves, light-demanding non-evergreen species, such as *Q. pubescens*, are tendentially more drought tolerant than shade-tolerant species, while the opposite was observed for evergreen species (such as *Q. ilex*). In our study, where we applied different stress levels (VWC) with a species-specific threshold close to wilting point, the more xeric species *Q. pubescens* and *Q. ilex* [119,120,121] showed a much greater survival rate (about 50%, and >70% respectively, and up to 100% at recovery) than *Q. robur*. *Q. pubescens* had a higher re-sprouting capacity at recovery than *Q. ilex* (data not shown), indicating a high drought resistance [101,122,123] and resilience with a fast recovery of photosynthetic activity after re-watering [124]. The geographical distribution of *Q. pubescens* suggests an intermediate drought resistance (e.g., [74]); therefore, the performance under high water stress was unexpected. This result agrees with other physiological studies on oaks’ behavior [125,126], which explain the role of photoprotective mechanisms on the functionality of the photosynthetic apparatus during severe drought [77,124]. Instead, *Q. ilex* may face xylem embolism in stem and shoots that could prevent growth recovery after re-watering [127], a phenomenon particularly accentuated in larger seedling size or above-ground development [128].

We acknowledge that plant response in a semi-controlled environment does not necessarily translate to the performance of open-field performance of planted seedlings under drought [129,130], since in other studies, lower growth and photosynthesis rates have been found in potted plants, without changes in biomass ratios among plant parts as compared to open-field transplants [131]. Thus, our results should be confirmed with field trials where roots have an entire soil profile to develop.

## 4. Materials and Methods

### 4.1. Plant Material

In 2017, one-year-old seedlings of *Q. robur* (pedunculate oak), *Q. pubescens* (downy oak), and *Q. ilex* (holm oak)—were grown in containers (0.650 L) in a nursery in central Italy. We used two substrates, one traditional mixture made of 70% peat and 30% pumice, hereafter peat (*Pe*), and the other one made of pure coconut coir with 70% of pith (fine residual material) and 30% of fibers, hereafter coir (*Co*), and three different fertilization formula, applied in a solid form: (1) nursery standard, *St*, a controlled-release fertilizer (CRF) widely used in Italy to commercially grow oak seedlings (Osmocote Exact Standard), providing NPK rates per volume of 450.0, 270.0 and 330.0 mg/L of substrate, respectively, plus micronutrients at 3 kg·m^−3^; (2) nursery standard fertilization enriched in phosphorus (*P*), with NPK content per volume mg/L: N 450.0, P 690.0, K 330.0; (3) nursery standard enriched in potassium (*K*) with NPK content per volume mg/L: N 440.0, P 440.0, K 720.0. Substrates and fertilizations were factorially combined for a total of six stocktypes per species. Further details on substrates and fertilizations, as well as on nursery location and cultivation practices, are reported in [69], together with the first-year morpho-physiological response of the species to substrate and fertilization treatments. For the studied *Quercus* species, Mariotti et al. [69] reported that peat and K-enriched fertilization resulted in larger seedlings and slightly improved physiological responses, while seedlings grown in coir showed a proportionally similar root system development and fibrosity, and a lower shoot-to-root ratio than plants grown in peat.

One year later, in March 2018, 45 seedlings per stocktype and species (810 seedlings in total) were moved into a larger greenhouse (Pistoia, Italy, WGS 84 43°51′43.4″ N 10°58′49.2″ E, 85 m a.s.l.) and transplanted into 3 L black plastic containers (14 × 14 cm square top section, 21 cm of depth and round bottom), filled with pure coconut coir without any further fertilization. To prevent excessive heating, each pot was placed into a slightly bigger white container. The means of plant height at transplanting were QR: 57.6 ± 10.8 in *Pe*, 45.8 ± 10.3 in *Co*, and 48.3 ± 11.4 in *St*, 51.5 ± 12.2 in *P*, 55.4 ± 11.8 in *K*; QP: 36.8 ± 11.5 in *Pe*, 22.4 ± 8.2 in *Co*, and 27.9 ± 10.7 in *St*, 27.0 ± 11.1 in *P*, 33.9 ± 14.0 in *K*; QI: 52.7 ± 13.1 in *Pe*, 35.0 ± 10.3 in *Co*, and 40.9 ± 13.0 in *St*, 41.8 ± 13.3 in *P*, 48.9 ± 16.7 in *K*. Each seedling was irrigated by drip irrigation with two drippers (capacity: 60 mL min^−1^ per pot) placed at the two sides of the stem. Until the start of the water stress trial, seedlings were irrigated daily to maintain the substrate field capacity (pot capacity). The temperature inside the greenhouse was measured but not moderated.

### 4.2. Experimental Design

The water stress experiment was carried out between June and July 2018 for two weeks, from 27 June (T_0_) to 10 July (T_1_, end of the experiment). It was timed to simulate the onset of a drought period that commonly occurs in late spring or in early summer. During the trial, seedlings were irrigated daily according to three water regimes, as follows:

(a) No stress (control, *C*) was determined by maintaining pot capacity. Before the beginning of the trial, 5 pots per substrate and per species were saturated and weighted when no percolation was observed. The amount of water needed to maintain pot capacity by daily irrigation was calculated as difference after re-weighting the pots 24 h later.

(b) Medium stress (*MS*) was calculated as 50% of the daily water amount provided at pot capacity.

(c) High water stress (*HS*) was defined by maintaining a species-specific threshold of Volumetric Soil Water Content (VWC). VWC was targeted at 13%, 9%, and 6% (±1% tolerance) for QR, QP, and QI, respectively, to induce high water stress in the three oak species according to literature [115]. Before the beginning of the trial, five pots per substrate and per species were irrigated up to pot capacity and then left without any water supply; VWC was monitored twice a day until it reached the species-specific threshold when they were weighted. Daily irrigation to maintain the values was calculated by weighing the same pots 24 h later. We made preliminary tests to determine the time needed for each species to reach the defined VWC in the greenhouse (6 days for *Q. ilex*, 5 days for *Q. pubescens* and 3 days for *Q. robur*).

VWC was measured by a FieldScout TDR 150 (Spectrum Technologies, Aurora, IL, USA) and monitored twice a day during the experiment on five randomly selected pots for each substrate per fertilization combination per species and water stress treatment (270 containers in total). For each species, 45 seedlings per stocktype were placed along three irrigation lines (one per watering regime; 15 seedlings per line). Environmental conditions in the greenhouse, i.e., photosynthetically active radiation, air temperature, and humidity, were monitored before and throughout the experiment by a weather station (PCE-FWS 20, PCE Instruments, Capannori, Italy) and resulted in being homogenous across the greenhouse. From the end of the water stress experiment (T_1_), all seedlings were irrigated daily at pot capacity until the end of the growing season (Oct 2018).

### 4.3. Seedling Survival, Leaf Mortality, Plant Height, and Recovery after Water Stress Treatments Application

In each species, leaf status (alive or dead) was visually assessed at T_1_; at T_1_, a seedling was classified as “live”’ if some leaves were still green otherwise “dry” when all leaves were desiccated or abscised—i.e., but the plant could be still alive; seedling survival was confirmed by visually assessing the resprouting at the recovery (T_rec_), after two months of irrigation, at the beginning of September 2018. Thus, the data related to leaves and plant survival were analyzed only under *HS*, as the other two water regimes showed neither seedling nor leaf mortality.

Seedling height was measured on all plants at the beginning (T_0_), and at the end of the experiment (T_1_; H_0_ and H_1_, respectively). Height increments for each seedling resulting by difference and percent height increments between T_0_ and T_1_ (I%) were also calculated; I% was preferred to increment absolute value due to the high variability of the data and to the low values (measured after a fortnight).

### 4.4. Leaf Water Potential and Chlorophyll Fluorescence during Water Stress Experiment

For each species, predawn (Ψ_pd_) and midday (Ψ_min_) leaf water potentials were measured at T_0_ and T_1_ by using a PMS pressure chamber (PMS Instruments Co., Corvallis, OR, USA) on 4 plants randomly selected, 1 leaf per plant, per water regime and stocktype (total = 72 seedlings per species, 216 in total). Predawn measurements were obtained during the two hours before dawn (dawn between 5:15–5:30 a.m. LT), midday measurements between noon and 2:00 p.m., on three contiguous sunny days, one day per species.

Steady-state chlorophyll *a* fluorescence (PAM-2000, Walz, Germany) was measured at predawn and midday on the same days in the greenhouse and at saturating light at T_0_ and T_1_ on a total of 810 leaves marked before experiment onset (3 leaves per plant; 5 plants per water regime x stocktype). Fluorescence yield under saturating light was partitioned between photochemistry quantum yield (Φ_II_) and two competing non-productive pathways, which accounted for energy dissipation by downregulation (Φ_NPQ,_ regulated thermal non-photochemical dissipation) and other non-photochemical losses (Φ_NO_, constitutive non-light induced thermal dissipation); being the sum of Φ_II_, Φ_NPQ,_ and Φ_NO_ equal to 1 (equations in [132]).

The performance index on absorption basis (PI_ABS_) [133] was retrieved by the Plant Efficiency Analyser (Handy PEA, Hansatech, Norfolk, UK) at T_0_ and T_1_ between 8.00 and 10.00 a.m. after 40 min of dark adaptation, on 9 leaves per water regime × stocktype (1 leaf per plant on randomly selected plants; total = 162 leaves per species), in the same days of water potential measurements.

### 4.5. Statistical Analysis

Mortality and occurrence of seedlings with dry shoot at T_1_ under high stress were analyzed using a Mixed Generalized Linear Model based on a binomial distribution (live or dead) by glm() function in R library “stats” testing main factors (substrate and fertilization) and their interaction effect. Models with and without interaction term, that is y_mod1_~Sub×Fert vs y_mod2_ ~Sub + Fert were compared using Akaike information criterion (AIC_c_) corrected for samples size and number of parameters in the model [134] and the difference with the best model (Δ = AIC_c I_ − AIC_c minimum_). Chi-square (χ^2^) test with Yates correction and Bonferroni correction for multiple contrasts was also applied to highlight differences among the three fertilization treatments in case of a significant fertilization effect.

Physiological (leaf water potential) and growth (plant height) traits (expressed as mean values ± standard error) were analyzed by multifactorial ANOVA for a general linear model (GLM) with three fixed factors: water regime, substrate, and fertilization; Tukey was used as the post-hoc test (α ≤ 0.05). Assumptions were verified prior to ANOVA. When ANOVA assumptions were not met, data were analyzed by comparing bootstrapped mean values and confidence intervals (R library “boot”, runs = 20,000). Effects were considered significant when *p ≤ 0.05*. Data were analyzed using R software (R Core Team 2021).

## 5. Conclusions

Using semi-controlled conditions, our experiment helped to define the potential intrinsic post-planting responses of common Mediterranean oak species to early summer drought. Our results suggest that substrate and fertilization can influence water stress resistance in the studied *Quercus* species. The following key points emerged from our study:

- Contrary to our hypothesis, seedlings grown in coir performed equally or better than in peat in water stress response; this could be due to the lower shoot-to-root ratio of seedlings grown in this substrate. This can be an advance in the way of looking for sustainable alternatives for peat.
- Nursery fertilization with high *P* promoted survival under water stress conditions in the trial, probably related to the physiological benefit of higher tissue *P* concentration after nursery cultivation.
- K-enriched fertilization at the nursery stage resulted in inconsistent findings under high water stress during the following growing season, with greater mortality in *Q. robur*, greater survival in *Q. pubescens*, and no effects in *Q. ilex* as compared to the standard fertilization, and no relevant effects on height growth. The negative effect on *Q. robur* could be due to the lack of accumulation of this element in the tissues after nursery cultivation in combination with the bigger size of the nursery seedlings that might have suffered the limited volume explorable by the roots in the pot.
- *Q. pubescens*—despite being described as a species with an intermediate stress resistance in Mediterranean environments—showed high resistance and acclimation to severe water stress, comparable, or better than *Q. ilex*.

## Figures and Tables

**Table 1:** GLM model results (P values) for percentage of live seedlings (not dry) at T_1_ and survival at T_rec_ of *Quercus* species after transplanted in controlled conditions under high water stress. In bold significant results.

	Live Seedlings at T_1_			Seedlings Survival at T_rec_		
	*Q. robur*	*Q. pubescens*	*Q. ilex*	*Q. robur*	*Q. pubescens*	*Q. ilex*
Substrate	0.937	0.205	0.398	0.756	0.077	0.822
Fertilization	**0.036**	**0.000**	0.057	**0.020**	**0.000**	0.117
Sub × Fert	0.799	0.715	0.241	0.583	0.211	0.077

**Table 2:** Live seedlings at T_1_ (no dry shoot) and survival at T_rec_ (data in %) of *Quercus* species after transplanted in controlled conditions under high water stress. *Pe*, *Co*: peat and coir substrate; *St*, *P*, *K*: standard, P- and K-enriched fertilization. T_1_: end of the water stress experiment, T_rec_: recovery. * couples comparisons by Chi-square test performed when GLM model results for fertilization were significant.

		Live seedlings at T_1_	Seedlings survival at T_rec_
*Q. robur*	*Pe*	37.8	51.1
	*Co*	35.6	55.6
		
	*St*	36.7	63.3
	*P*	53.3	63.3
	*K*	20.0	33.3
		P > St and K *	P and St > K *
*Q. pubescens*	*Pe*	42.2	77.8
	*Co*	55.6	91.1
		
	*St*	20.0	66.7
	*P*	30.0	86.7
	*K*	96.7	100
		K > P and St *	K and P > St *
*Q. ilex*	*Pe*	48.9	66.7
	*Co*	57.8	68.9
		
	*St*	36.7	53.3
	*P*	66.7	76.7
	*K*	56.7	73.3

**Table 3:** Multifactorial ANOVA test results for seedlings height (cm) at T_0_ (before the onset of water stress). Lowercase letters indicate homogeneous groups (*p* ≤ 0.05). *Pe*, *Co*: peat and coir substrate; *St*, *P*, *K*: standard, P-and K-enriched fertilization. In bold significant results.

	*Q. robur*	*Q. pubescens*	*Q. ilex*
*Co*	57.3 a	33.2 a	58.0 a
*Pe*	72.1 b	48.5 b	66.2 b
	***p < 0.0001***	***p < 0.0001***	***p < 0.0001***
*K*	68.3 b	45.9 b	61.9
*P*	64.9 ab	38.3 a	60.5
*St*	61.0 a	38.5 a	64.0
	***p = 0.029***	***p = 0.001***	*p = 0.208*
*Sub × Fert*	*p = 0.358*	*p = 0.593*	*p = 0.166*

**Table 4:** Multifactorial ANOVA test results for performance index PI_ABS_ (mean ± SE) at T_0_ and T_1_. Lowercase letters indicate homogeneous groups (*p ≤ 0.05*). Levels: *C*, *HS*: control and high water stress; *Pe*, *Co*: peat and coir substrate; *St*, *P*, *K*: standard, P- and K-enriched fertilization.

		T_0_		T_1_	
*Q. robur*	*C*	3.61 ± 0.31		4.94 ± 0.27	b
	*HS*	4.73 ± 0.29		3.37 ± 0.29	a
				
	*Pe*	3.98 ± 0.30		4.48 ± 0.27	
	*Co*	4.34 ± 0.31		3.86 ± 0.33	
				
	*St*	4.63 ± 0.40	b	4.60 ± 0.41	
	*P*	3.52 ± 0.29	a	3.85 ± 0.29	
	*K*	4.32 ± 0.40	a	4.08 ± 0.40	
*Q. pubescens*	*C*	7.11 ± 0.57		9.06 ± 0.61	
	*HS*	6.93 ± 0.82		8.59 ± 0.64	
				
	*Pe*	5.67 ± 0.58	a	8.00 ± 0.61	a
	*Co*	8.06 ± 0.68	b	9.62 ± 0.64	b
				
	*St*	7.02 ± 0.95		8.17 ± 0.93	
	*P*	5.87 ± 0.66		9.16 ± 0.69	
	*K*	8.04 ± 0.83		8.96 ± 0.73	
*Q. ilex*	*C*	10.08 ± 0.73		7.50 ± 0.45	b
	*HS*	9.54 ± 0.75		4.04 ± 0.41	a
				
	*Pe*	9.09 ± 0.65		5.51 ± 0.45	
	*Co*	10.42 ± 0.79		6.13 ± 0.53	
				
	*St*	12.95 ± 0.91	b	5.58 ± 0.57	
	*P*	9.25 ± 0.90	a	6.15 ± 0.70	
	*K*	7.45 ± 0.62	a	5.74 ± 0.55	

## Data Availability

Not applicable.
